# Functional Gene-Expression Analysis Shows Involvement of Schizophrenia-Relevant Pathways in Patients with 22q11 Deletion Syndrome

**DOI:** 10.1371/journal.pone.0033473

**Published:** 2012-03-22

**Authors:** Nico J. M. van Beveren, Lianne C. Krab, Sigrid Swagemakers, Gabriella Buitendijk, Erik Boot, Peter van der Spek, Ype Elgersma, Therese A. M. J. van Amelsvoort

**Affiliations:** 1 Department of Psychiatry, Erasmus University Medical Centre, Rotterdam, The Netherlands; 2 Department of Neuroscience, Erasmus University Medical Centre, Rotterdam, The Netherlands; 3 Department of Bioinformatics, Erasmus University Medical Centre, Rotterdam, The Netherlands; 4 Mondriaan Mental Health Care, Heerlen, The Netherlands; 5 Department of Psychiatry and Psychology, Maastricht University, Maastricht, The Netherlands; 6 Delta Center for Mental Health Care, Rotterdam, The Netherlands; 7 Virenze Mental Health Care, Gronsveld, The Netherlands; 8 Department of Psychiatry, Academic Medical Centre, Amsterdam, The Netherlands; Rikagaku Kenkyūsho Brain Science Institute, Japan

## Abstract

22q11 Deletion Syndrome (22q11DS) is associated with dysmorphology and a high prevalence of schizophrenia-like symptoms. Several genes located on chromosome 22q11 have been linked to schizophrenia. The deletion is thought to disrupt the expression of multiple genes involved in maturation and development of neurons and neuronal circuits, and neurotransmission. We investigated whole-genome gene expression of Peripheral Blood Mononuclear Cells (PBMC's) of 8 22q11DS patients and 8 age- and gender-matched controls, to (1) investigate the expression levels of 22q11 genes and (2) to investigate whether 22q11 genes participate in functional genetic networks relevant to schizophrenia. Functional relationships between genes differentially expressed in patients (as identified by Locally Adaptive Statistical procedure (LAP) or satisfying p<0.05 and fold-change >1.5) were investigated with the Ingenuity Pathways Analysis (IPA). 14 samples (7 patients, 7 controls) passed quality controls. LAP identified 29 deregulated genes. Pathway analysis showed 262 transcripts differentially expressed between patients and controls. Functional pathways most disturbed were cell death, cell morphology, cellular assembly and organization, and cell-to-cell signaling. In addition, 10 canonical pathways were identified, among which the signal pathways for Natural Killer-cells, neurotrophin/Trk, neuregulin, axonal guidance, and Huntington's disease. Our findings support the use of 22q11DS as a research model for schizophrenia. We identified decreased expression of several genes (among which COMT, Ufd1L, PCQAP, and GNB1L) previously linked to schizophrenia as well as involvement of signaling pathways relevant to schizophrenia, of which Neurotrophin/Trk and neuregulin signaling seems to be especially notable.

## Introduction

Deletions of chromosome 22q11 are associated with a high prevalence of dysmorphology (velo-cardio-facial syndrome; 22q11DS), cognitive and behavioural disorders and schizophrenia-like psychosis [1], [2]. It is thought that a hemizygous 22q11 deletion disrupts the expression of multiple genes involved in maturation, development of neurons and neuronal circuits, and neurotransmission [3]. Several genes located in the 22q11 region have been linked to schizophrenia, including COMT, ProDH, Ufd1L, PCQAP [4], and, recently, GNB1L [5]. It is likely that genes present in the 22q11 deleted region form functional networks (i.e. metabolic-and signaling pathways) with other genes outside this region involved in development and neuronal functioning, and that the decreased expression of 22q11 genes alters the functional activity of these pathways. It is however unclear which pathways these are, and what their relationship is with pathways involved in idiopathic schizophrenia. More insight into the nature of these functional networks may increase our understanding in the genetic networks involved in the developmental, psychiatric, cognitive and behavioural disturbances seen in 22q11DS including schizophrenia. Moreover, it will provide more understanding of the relationship between 22q11DS and schizophrenia and the value of 22q11DS for investigating schizophrenia pathology.

The combination of gene expression profiling and biomics is proven to be a powerful technology to identify functional changes in genetic networks [6]. However, for neuropsychiatric disorders a major limitation is the inaccessibility of the living brain and the heterogeneity of many psychiatric disorders. This is especially relevant for disorders such as schizophrenia, which show a prolonged life-long course during which the effects of normal and abnormal brain development, drug abuse and medication influence the results of post-mortem gene-expression studies [6], [7]. Peripheral Blood Mononuclear Cells (PBMC's) express many brain relevant genes [8] and have been suggested as ‘window-on-the brain’ surrogate tissue to investigate neuropsychiatric disorders. In schizophrenia, a number of recent studies have explored the use of various types of PBMC's [9]–[13]. The key assumption of these studies is that although peripheral blood cells in schizophrenia show no clear aberrations in function or morphology, subtle molecular-biological aberrations are present and can be informative about the molecular-biological underpinnings of schizophrenia. However, in non-22q11DS schizophrenia a multivariate genetic and environmental cause is suspected. Therefore PBMC gene expression of patients with idiopathic schizophrenia may be largely variable. We reasoned that in 22q11DS the deletion will most likely result in decreased PBMC expression of 22q11 genes which may in turn influence the expression of other genes outside the deleted region more robustly and persistently than in idiopathic schizophrenia. Furthermore, using functional pathway analysis, 22q11DS might offer a view of the concerted altered expression of genes both within and without the 22q11 deleted region that may alter the activity of functional genetic networks.

PBMC gene expression in 22q11DS patients has not yet been investigated, neither the individual expression levels of genes in the 22q11 deleted region, nor the influence of the deletion on functional genetic pathways. Thus, we investigated gene expression in PBMC's of 22q11DS patients, with the objective to (1) investigate the expression levels of 22q11 genes and to (2) investigate whether 22q11 genes, together with genes outside this region, participate in functional networks relevant for (neuro)developmental abnormalities in 22q11DS.

## Materials and Methods

### Subjects

8 22q11DS patients and 8 age-and gender matched controls were included in this study. Deletions at chromosome 22q11 were previously identified in all subjects by Fluorescence In-Situ Hybridization (FISH). This study was approved by the institutional review boards of the two participating centres (Erasmus University Medical Centre, Rotterdam, and Academic Medical Centre, Amsterdam) and was performed in accordance with the declaration of Helsinki. All subjects provided written informed consent.

### Samples

From each participant 30 ml of blood was drawn into heparinized tubes. PBMC's were isolated by Ficoll-gradient separation starting 90 minutes after the drawing of blood. Cells were subsequently disrupted (Qiashredder kit; Qiagen), and RNA was isolated (RNeasy minikit; Qiagen) with an additional DNAse digestion step (RNase-free DNase set; Qiagen), all according to the manufacturer's protocol, diluted in nuclease free water and frozen at −80 C before use. After thawing the isolated RNA was biotinylated into cRNA using the One-Cycle Target Labeling and Control Reagents Kit (Affymetric Co) according to the manufacturer's protocol. Before hybridization RNA quality and integrity was assessed using the Agilent 2100 BioAnalyzer (Agilent) and RNA purity and quantity with the NanoDrop ND-1000 spectrophotometer (NanoDrop Technologies). Biotinylated cRNA was hybridized to the Affymetrix Human Genome U133 plus 2.0 GeneChip© microarray containing 54,675 probe sets (Affymetrix Co). Each sample was individually biotinylated and hybridized to an individual microarray. Biotinylation was performed in two batches with randomization of samples across the batches. Hybridization was performed in one batch. The arrays were scanned and analyzed using Affymetrix Microarray Suite 4.2 software.

### Statistical analysis

Probe sets that were absent in all samples (according to Affymetrix software) were omitted from further analysis. Raw intensities (33,196 probe sets) were normalized by quantile normalization. Data analysis was done using OmniViz version 5.0 (Biowisdom) and R program. Minimum thresholds were set at 30. To investigate the expression levels of genes in the 22q11 region between patients and controls we applied two separate methods, Significance Analysis of Microarrays (SAM) [14] (OmniViz) and Locally Adaptive Statistical procedure (LAP) [15] (R program). SAM uses permutations of the repeated measurements of the expression levels to estimate the false discovery rate (FDR). LAP combines a FDR approach with information about chromosomal location of the genes and is specifically suited to identify differentially expressed regions that are involved in known chromosomal aberrations [15]. It should be noted that LAP may identify significantly decreased expression of genes which are actually not expressed at all, due to way the LAP algorithm uses information about chromosomal location. These not expressed genes were removed from further analyses. To investigate the functional relationships between genes which are differentially expressed between patients and controls we used the Ingenuity Pathway Analysis (IPA) (Ingenuity Systems®; www.ingenuity.com; October 2007). The IPA is an internet-accessible database in which knowledge about the interaction between genes and gene products is stored based on known interactions in the literature [16]. Thus, the IPA defines genetic networks, functions, and metabolic-and signaling pathways which describe functional relationships between gene products and presents as output networks, functions and metabolic-and signaling pathways in which the genes in the dataset participate more than can be expected by change. It does so by calculating a significance score (using the right-tailed Fisher's exact test and expressed as a p-value) for each process by computing the number of deregulated genes that participate in a network or pathway relative to the total number of occurrences of these genes in all functional/pathway annotations stored in the IPA. The significance value associated with networks and pathways is a measure for how likely it is that genes from a dataset participate in that function or pathway. For genetic networks an IPA score> = 3 is considered significant, for canonical pathways a p-value<.05 is considered significant. We uploaded into the IPA a dataset which consisted of the genes identified by LAP and of genes with different expression levels between patients and controls according to the criteria: t-test between patients and controls significant (p<0.05) and a 1.5 or more fold-change (FC) in expression level in patients compared to controls.

## Results

### Subjects

8 22q11DS patients and 8 controls were included. Four patients had a previous history of psychotic symptoms, whereas four did not (further characteristics see **table 1**).

**Table 1:** Subject characteristics.

Pair #	PATIENTS				CONTROLS	
	Psychosis	Antipsychotic use	Age	gender	Age	gender
1	No	none	18	F	19	F
2	No	none	39	F	39	F
3	No	none	25	M	25	M
4	No	none	17	F	18	F
5	Yes	zuclopentixole	27	M	27	M
6	yes*	risperidone	32	F	30*	F
7	Yes	risperidone	25	M	26	M
8	Yes	risperidone	21	M	21	M

* excluded cases (N = 2) because of bad signal-to-noise ratio of the expression data; coincidentally, the excluded cases form a matched pair.

Routine quality controls of the gene expression results showed sub-optimal signal-to-noise ratios in two subjects (one patient with psychosis and one control). These two samples were excluded from further analyses.

### Global significance analysis of gene expression

SAM showed 14 transcripts differentially expressed between patients and controls (False Discovery Rate (FDR) = 6.6%; falsely called<1) (see **table 2**). Ten of these transcripts are located in the 22q11 region.

**Table 2:** Significance Analysis of Microarrays (SAM).

Probe Set ID	Gene Title	Gene Symbol	Entrez Gene	Chromosomal Location
229906_at	Armadillo repeat containing 7	ARMC7	79637	chr17q25.1
217427_s_at	HIR histone cell cycle regulation defective homolog A (S. cerevisae)	HIRA	7290	chr22q11.2| chr22q11.21
1553974_at	Hypothetical protein 1	LOC128977	128977	chr22q11.2
202483_s_at	RAN binding protein 1	RANBP1	5902	chr22q11.2
203152_at	Mitochondrial ribosomal protein L40	MRPL40	64976	chr22q11.2
209103_s_at	Ubiquitin fusion degradation 1 like (yeast)	UFD1L	7353	chr22q11.2
210010_s_at	Solute carrier family 25 (mitochondrial carrier; citrate transporter), member 1	SLC25A1	6576	chr22q11.2
32032_at	DiGeorge syndrome critical region gene 14	DGCR14	8220	chr22q11.2| chr22q11.2
208818_s_at	Catechol-O-methyltransferase	COMT	1312	chr22q11.2-q11.23
206184_at	v-crk sarcoma virus CT10 oncogene homolog (avian)-like	CRKL	1399	chr22q11.2| chr22q11.21
212180_at	v-crk sarcoma virus CT10 oncogene homolog (avian)-like	CRKL	1399	chr22q11.2| chr22q11.2
202206_at	ADP-ribosylation factor-like 4C	ARL4C	10123	chr2q37.1
235289_at	Eukaryotic translation initiation factor 5A2	EIF5A2	56648	chr3q26.2
230685_at	Hypothetical protein LOC644873	FLJ33630	644873	chr5q23.3

**Figure 1:**
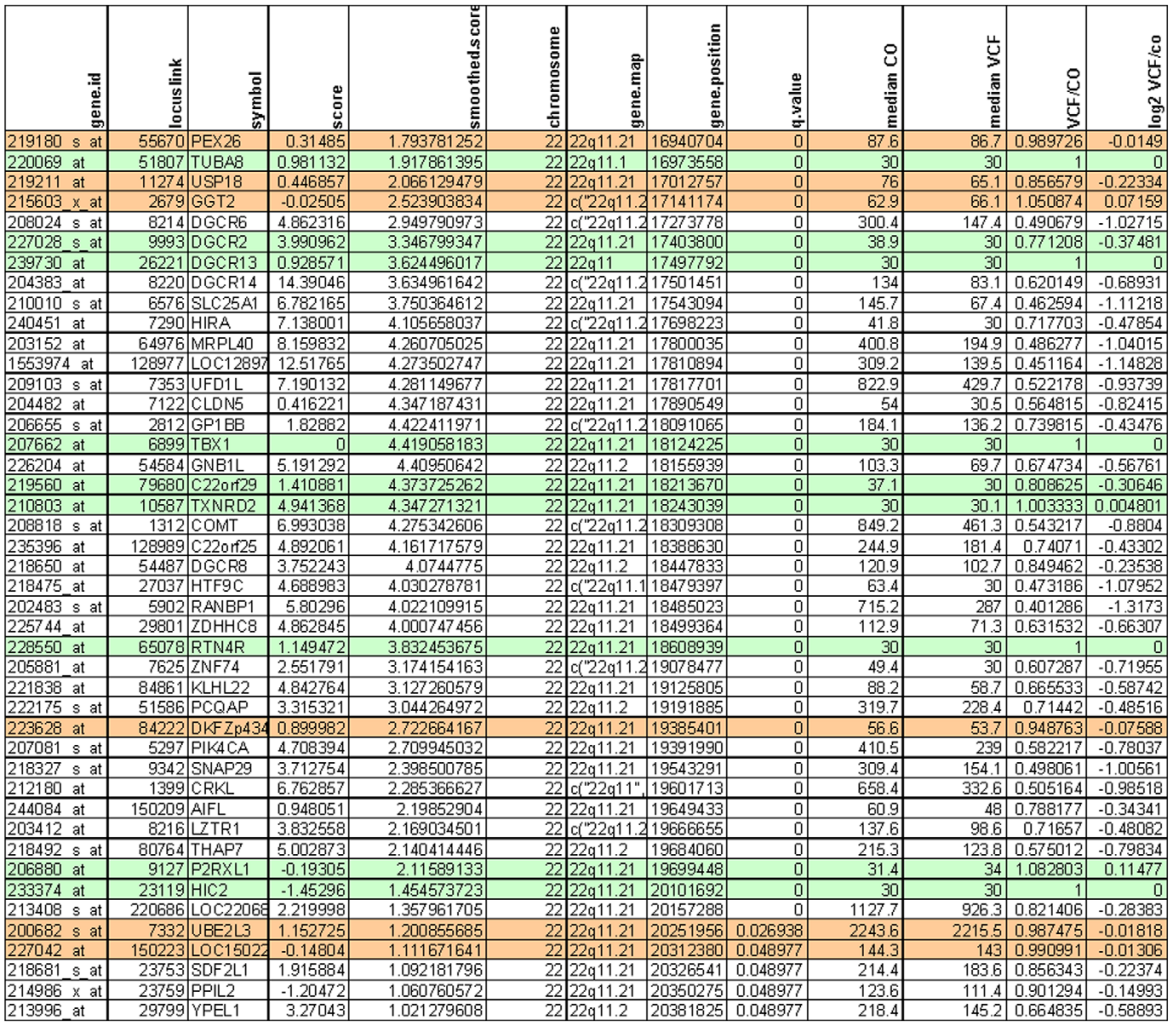
Results of the Locally Adaptive Statistical Procedure (LAP). Green rows show genes which are not expressed in PBMC's; red rows show genes not differentially expressed in PBMC's between patients and controls. Columms respectively show (from left to right): Probe Set ID; Entrez Gene ID; Gene Symbol; SAM score (*d_i_*); smoothed score (*S_i_*); chromosomal localization and position; qvalues; median intensity of the controls (CO); median intensity of the 22q11DS patients; ratio 22q11DS/CO and log2 ratio 22q11DS/CO.

LAP identified 44 genes (p value<0.05), all in the 22q11 region (see **figures 1** **and** **2**).

**Figure 2:**
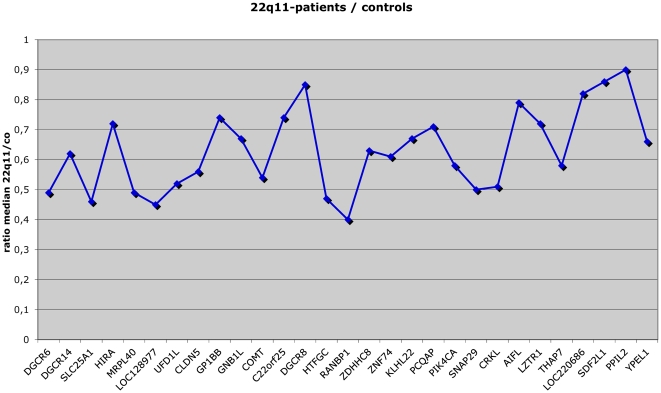
Expression levels of genes in the 22q11 deleted region. The figure shows the ratio of the median expression levels of patients versus controls for each of the genes shown along the x-axis.

Of these 44 genes, 9 were not expressed in blood and 6 were not differentially expressed between patients and controls, resulting in 29 differentially expressed genes.

### Pathway analysis

The criteria p<0.05 and FC>1.5 showed 262 transcripts differentially expressed between patients and controls. These 262 transcripts are described in **table S1**. Combining these with the genes identified by LAP (n = 29) and uploading these in the IPA generated 128 genes available for building networks and investigating relationships. Seven functional networks are considered to be significantly associated with the uploaded gene set according to IPA criteria (IPA score>3) These networks are involved in (among others) cardiovascular disease, cardiovascular system development and function, nervous system development and function, connective tissue development and function, cell signaling, and cell-to-cell signaling and interaction. **Table 3** shows the seven highest ranking networks, and the biological processes associated with these networks.

**Table 3:** Significantly deregulated genetic networks as identified by the Ingenuity Pathways Analysis (IPA).

ID	Molecules in Network	Score	# of Focus Molecules	Top Functions
1	ARNT2, B2M, ↑**BASP1**, ↓**CDC42**, CYBA, H2-Q1, HLA-E, JAK2, ↑**KCNJ15**, KLRC1, ↓**KLRC2**, ↓**KLRC3**, ↓**KLRD1**, ↑**MEF2C**, ↑**MXD1**, MYCN, PAK1, ↓**PARD6A**, PARD6G, ↓**PIK3R3**, ↑**PPM1F**, ↓**PTPN4**, QDM, RAC2, ↓**RANBP1**, Rb, RB1, RBL2, ↓**SEPT5**, ↓**SNAP29**, ↑**SNX5**, STX1A, ↑**STXBP2**, ↑**TLR4**, VAMP2	26	18	Cell Cycle, Connective Tissue Development and Function, Nervous System Development and Function
2	↓**ACVR2B**, ↓**CD2**, ↓**COMT**, CRK, ↓**CRKL**, ↓**CXCL10**, ↑**DDEF1**, ↑**DOCK4**, E2F4, EPHB6, ESR1, ↑**ETV6**, ↓**EXOSC2**, EXOSC7, EXOSC8, EXOSC9, EXOSC10, ↑**HSF1**, Hsp70, HSPA7, ↑**MAFG**, MAP4K5, MYC, NFE2L3, ↓**NQO1**, ↑**P2RX1**, ↑**PCID1**, PIK3R1, PLAG1, ↓**PPP2R2B**, RICS, ↑**SENP6**, ↑**SLC11A1**, SUMO1, XRN2	24	17	Cancer, Hematological Disease, Cell-To-Cell Signaling and Interaction
3	APEX1, ATF3, CARM1, ↓**CCL5**, CCR4, ↑**CD24**, CD63, CKM, ↓**CPT1A**, ↓**DGCR6**, EP300, ↑**GK**, ↑**HK2**, MDM2 (includes EG:4193), ↓**MYO6**, NEUROG3, ↑**OLIG1**, ↓**PIK4CA**, PPARGC1A, ↓**PRF1**, PTGDS, ↓**PYHIN1**, RELA, SELP, SLC2A4, ↑**SLC2A5**, ↓**TBX21**, TFDP1, ↑**TNFRSF10C**, TP53, TRIM28, TSC22D3, ZBTB17, ↓**ZDHHC8**, ↓**ZNF74**	22	16	Gene Expression, Cancer, Respiratory Disease
4	↑**ABCA1**, ↓**API5**, CDKN2A, CKB, ↑**CR1**, CR2, ↓**4S234E**, ↓**DGCR14**, DLG4, ↓**F2R**, FGF2, FLT1, ↓**GZMA**, HMGB2, ↑**HPSE**, Hsp27, KCNJ4, KCNJ12, KPNB1, LAMA1, ↑**LIN7A**, ↑**NAB2**, ↓**PCQAP**, RNA polymerase II, ↓**RNGTT**, RNMT, SMAD3, Smad2/3, SP1, STX12, SUPT5H, ↑**TFE3**, ↓**UFD1L**, ↓**ZNF83**, ↑**ZNF451**	22	16	RNA Post-Transcriptional Modification, Cell Signaling, Cardiovascular System Development and Function
5	↑**ADAM9 (includes EG:8754)**, APOB, ↓**APOBEC3G**, ↓**ARL4C**, CEBPA, CIAA1, CIAA2, COL10A1, COL11A2, COL18A1, COL1A1, COL2A1, COL3A1, ↓**COL5A3**, ↑**CSF2RA**, EGR2, GRM5, ↑**HES7**, HMGB2, Homer, HOMER1, **HOMER3**, ↑**HP**, ITGA2, ITGA9 (includes EG:3680), ITGB1, ITPR, NFYB, NFYC, ↑**PTPRE**, SHANK3, ↑**THBD**, ↑**TNFRSF8**, ↓**TXNRD2**, ↑**ZBTB7B**	17	13	Dermatological Diseases and Conditions, Cardiovascular Disease, Cell Cycle
6	↑**AQP9**, ARF1, CARM1, CBX1, CBX5, ↑**CD53**, **↓CENTG2**, **↓CHAF1A**, Creb, ↑**CREB5**, CREM, ↑**ERAF**, GRIA2, GRM7, H3F3B, **↓HIRA**, HIRIP3, Histone h3, KIR3DL1, MBD1, NCAPD2, NCOA1, NCOA2, PICK1, Pkc(s), PLD1, ↑**PRKCA**, **↓RORA**, **↓RORC (includes EG:6097)**, SETDB1, SFTPA1, **↓SMC2**, SMC4, **↓THAP7**, TRIM24	15	12	Cell-To-Cell Signaling and Interaction, Nervous System Development and Function, Cell Morphology
7	↑**ACSL4**, Actin, Arp2/3, ↑**BACH2 (includes EG:60468)**, **↓C19ORF12**, CAM, CD44, **↓CLDN5**, **↓CLDN19**, **↓CLIC5**, DNMBP, HDAC4, MARK4 (includes EG:57787), NCOR2, PARD3, **↓PARD6A**, PARD6B, PARD6G, ↑**PDK1**, PFN1, **↓PHLDB2**, PKC (λ,ζ), PRKCI, ↑**RAPGEF2**, SMARCA4, STK11, TJP1, TJP2, TJP3, VIM, WAS, ↑**WASF1**, WASF2, YWHAQ, YWHAZ	13	11	Cell Morphology, Cellular Development, Cell-To-Cell Signaling and Interaction

The table displays the seven functional networks identified by the IPA as significantly (score>3) associated with the geneset defined by the two critera (1) deregulated between patients and controls according to LAP or (2) t-test<0.05 and Fold Change>1.5. The table displays the genes associated with the functional networks (genes which are present in the uploaded gene set are in bold). The arrows behind a gene indicate the direction of change (arrow pointing upwards: increased expression in patients, arrow pointing downwards: decreased expression in patients).The column ‘score’ gives the significance score for the network. The column ‘# of genes’ gives the number of genes in the network. Each network consists –by definition of the IPA- of 35 genes. The last column shows the functions in which each network is predominantly involved.

Canonical pathways identified by the IPA in which the uploaded gene set participates more than can be expected by chance (p<.05) are Natural Killer (NK)-cell signaling (p = .0004), neurotrophin/Trk signaling (p = .003), Fibroblast Growth Factor (FGF) signaling (p = .006), leukocyte extravasation signaling (p = .007), neuregulin signaling (p = .002), complement and coagulation cascades (p = .002), Platelet Derived Growth Factor (PDGF) signaling (p = .003), ERK/MAPK signaling (p = .003), axonal guidance signaling (p = .047), and Huntington's disease signaling (p = .049).

### Comparison of PBMC expression levels with brain expression levels in mouse models

The Df1/+ heterozygous mice (Df1/+), a model for 22q11DS, displays specific deficits in hippocampus-dependent functions. Sivagnanasundaram et al [17] analyzed the hippocampal gene expression of genes mapping to the deleted region as compared to wild type (WT) mice. Twelve genes were differentially expressed in the hippocampus. Of these twelve genes seven were also expressed in our PBMC samples (DGCR6, RANBP1, ZDHHC8, HTF9C, COMT, CLDN5, and UFD1L). The relative expression levels of these genes (hippocampal levels of Df1/+ vs WT mice (after Sivagnanasundaram et al [17]) and PBMC levels of 22q11DS patients vs controls) are shown in **table** 4.

**Table 4:** Relative hippocampal gene expression levels.

Gene	Relative hippocampal expression Df1/+ vs WT	Relative PBMC expression 22q11DS patients vs controls
DGCR6	0.45	0.49
RANBP1	0.53	0.40
ZDHHC8	0.86	0.63
HTF9C	0.63	0.47
COMT	0.54	0.54
CLDN5	0.68	0.56
UFD1L	0.78	0.52

The table dispays the relative hippocampal expression levels of Df1/+ vs WT mice (after Sivagnanasundaram et al, 2007 [17]) and PBMC gene expression levels of 22q11DS patients vs controls (this study) for seven genes expressed in both samples (mice and humans).

The relative expression levels of mice and humans correlated strongly and significantly (r: 0.677, p = 0.05, one sided). In another study, Meechan et al [18] investigated the expression levels of nine 22q11 orthologues in a 22q11DS mouse model (IDD, PRODH2, ZDHHC8, RANBP1, T10, COMT-MB, TBX1, UFD1L, and HIRA) in the developing and adult mouse brain. They also found diminished expression for the entire set of orthologues. Their findings indicated a fairly consistent decrease in expression levels with a magnitude between 40% and 60%, quite similar to our PBMC findings. Taken together, these results in mice cautiously suggest that diminished expression levels of 22q11 genes in PBMC's in humans might reflect decreased expression levels in the mouse brain for those genes which are also expressed in neuronal tissue.

## Discussion

To our best knowledge our study is the first to examine gene expression of 22q11DS patients. We show decreased expression of several genes present in the 22q11 deleted region. Among these are the genes which have been previously associated with schizophrenia, COMT, Ufd1L, PCQAP [4], and GNB1L [5]. Canonical pathway analyses show the significant involvement of the canonical pathways NK-cell signaling, neurotrophin/Trk signaling, FGF signaling, leukocyte extravasation signaling, neuregulin signaling, complement and coagulation cascades, PDGF signaling, ERK/MAPK signaling, axonal guidance signaling, and Huntington's disease signaling. The phenotypical expression of 22q11DS is highly variable. Among the major phenotypic features encountered are, apart from schizophrenia, cardiac anomalies, immunodeficiencies, craniofacial defects, hypocalcaemia, as well as a broad range of other psychiatric, cognitive and behavioral problems [19]. The most significantly involved canonical pathway in our study is NK-cell signaling. Immunodeficiency is one of the key features of 22q1DS, occurring secondary to thymic aplasia or hypoplasia with subsequent impaired thymocyte development. The majority of patients present with low or absent T-lymphocytes, but with spared B-lymphocytes and NK cells. An increased proportion of NK-cells have been described in patients with 22q11DS [20]–[22].However, the spectrum of severity of the immune deficit varies widely and does not appear to be related to other phenotypic abnormalities [23].Altered NK-cell activity in idiopathic schizophrenia has been reported in some studies [24] and is hypothesized to be related to the altered immunity and the reduced occurrence of autoimmune diseases and malignancies that has been observed in schizophrenia [25]. However, generally there is no clear agreement among studies on the involvement of NK cell activity in schizophrenia, with some studies showing lower activity, some higher activity, and the majority no change in activity between schizophrenia patients and controls [24]. Alterations in NK signaling as observed in our patient sample may be related to these previously reported immune deficits; however, it should be kept in mind that the finding of immune perturbations could also be a confounding epiphenomenon of the specific tissue under investigation.

There is far more support for the involvement of neurotrophin/Trk signaling in schizophrenia. This pathway is activated via the Trk family of receptors by various neurotrophic factors. The neurotrophic factors best known for their association with schizophrenia are Brain-derived Neurotrophic Factor (BDNF), Nerve Growth Factor (NGF), and neurotrophin-3 (NT-3). BDNF, NGF, and NT-3 are involved in a range of neuroplastic processes. Disturbed neurotrophin signaling is thought to underlie the neurodevelopmental disturbances seen in schizophrenia. Altered brain-(reviewed in Shoval and Weizmann [26]) and serum levels (reviewed in van Beveren et al ([27]) of neurotrophins have repeatedly been reported in schizophrenia.

There is also considerable support for an association of neuregulin with schizophrenia. Neuregulin is considered one of the genes most strongly associated with schizophrenia [28]–[30]. Neuregulin has been implicated in neuronal differentiation and migration. However, no consistent changes in neuregulin expression have been detected in schizophrenia. A recent report has shown that genetic variation in neuregulin (NRG1) is associated with variance in white matter brain content in normal individuals [31], as investigated by magnetic resonance imaging. Barnes et al [31] showed that genetic variation in the neuregulin variant SNP8NRG243177 is associated with variation in frontal brain structure in both grey and white matter. Reduced global white matter, most prominently in posterior and temporal regions, has been reported in 22q11DS [32]–[34]. So, altered neuregulin signaling as observed in our 22q11DS sample may be related to dysmaturation of white matter. Disturbances in axonal guidance signaling and Huntington's disease signaling are at present not specifically associated with schizophrenia but may underlie the general cognitive and behavioural disturbances found in schizophrenia. Moreover, in 22q11DS frequently motor disturbances, obsessive-compulsive symptoms, tics, and neurological aberrations are being observed [25], which are also found in Huntington's disease, and recent studies suggest that cognitive deterioration in a 22q11DS may already start in childhood [35], with a dramatic cognitive decline in adulthood in some psychotic 22q11DS patients.

There is an interesting possible relationship between 22q11DS and FGF signaling. Aggarwal et al [36] and Guo et al [37] described epistatic relationships between the FGF system and Tbx1. It is firmly established that Tbx1 is responsible for most of the congenital defects seen in 22q11DS patients and mouse models [38]–[40]. Tbx1 point mutations were found in patients who displayed the key physical symptoms of the classic 22q11DS phenotype, but did not have a 22q11 deletion [41]. There is incidental evidence for an association of FGF signaling with idiopathic schizophrenia. One publication reports decreased serum levels of FGF in schizophrenia [42], [43]. We could not identify reports describing involvement of PDGF signaling in schizophrenia. However, both FGF and PDGF are important growth factors of which involvement in schizophrenia has been suggested on theoretical grounds [30]. Disturbances in leucocyte extravasation signaling, and complement and coagulation cascades cannot be clearly related with 22q11DS and schizophrenia pathology. It has been postulated that subtle aberrations in cellular machinery may be present in all organs and cell types in schizophrenia [44], but only give rise to overt pathology in the nervous system. Such subtle aberrations may however become present in in-vitro situations, explaining differences in coagulation and leucocyte functions between patients and controls.

Taken together, our pathway analyses show a number of pathways previously associated with idiopathic schizophrenia among the pathways significantly associated with the deregulated gene set in our 22q11DS sample. Most notably for their involvement in schizophrenia are neurotrophin/Trk signaling and neuregulin signaling. Our findings cautiously suggest that the molecular-biological underpinnings of the psychotic phenomena observed in 22q11DS are at least partly related with those seen in ‘regular’ schizophrenia. Moreover, our findings are present even though only three patients show psychotic phenomena, and four do not. This suggests that molecular-biological pathways involved schizophrenia are deregulated in 22q11DS patients both with and without psychosis, and that, like in idiopathic schizophrenia, environmental factors modulate the expression of the psychosis phenotype in 22q11DS. Furthermore, the psychiatric phenotype in 22q11DS is extremely variable, and besides schizophrenia a range of other (neuro)psychiatric disorders, including autism spectrum disorders, mood disorder, attention deficit hyperactivity disorder, developmental delay, and learning disabilities. The co-occurrence of various psychiatric phenomena in 22q11DS may be an example where a general genetic vulnerability for mental impediments leads to variable phenotypic expression, probably dependent on (time-critical) environmental and stochastic influences. It is tempting to ascribe the other mental disorders present in 22q11DS to alterations in signaling pathways related to neuronal functioning, and/or pathways known to be disturbed in neuropsychiatric syndromes, such as axonal guidance signalling and Huntington's disease signalling, neuregulin signalling, and neurotrophin signalling. One may speculate that in the 22q11DS alteration in several distinct functional signaling cascades are present early on in life, with alterations in these cascades becoming more or less prominent, depending on environmental influences, and epistatic interaction with present or absent variants of risk genes outside the 22q11 deleted region.

Our study has a number of limitations; first, the number of subjects is small and heterogeneous (i.e. with-and without psychosis). Second: to identify functional networks we used a liberal statistical approach by not correcting for repeated measurements to obtain the gene set with criteria p<0.05 and FC>1.5, with a risk of identifying false-positives. However, we assume that combining this set with the genes present in the 22q11 deleted region (the set identified by LAP), which are certainly no false-positives, and investigating the functional relationships of combined gene set will ‘pull out’ the true positive genes as those will be the ones that predominantly have functional relationships with the 22q11 genes. Not so much a limitation as well as a caveat is that the approach presented here (investigating PBMC gene expression to gain insight in neuropsychiatric phenomena) relies on the assumption that molecular-biological aberrations observed in peripheral tissue can be informative about brain molecular-biological processes. Though may signaling pathways are similar in peripheral tissue and brain, and there are some reports that this approach is feasible [8], [45], the precise validity of this assumption remains to be further elucidated. In summary, this study (1) shows decreased expression of genes in the 22q11 deleted region in PBMC's of 22q11DS patients and (2) suggests the presence of deregulated signaling pathways relevant for schizophrenia pathology in PBMC's of 22q11DS patients. Generally, our findings support the use of the 22q11DS as a suitable, more homogeneous research model for schizophrenia. A prominent feature of both 22q11DS and schizophrenia is that they have a clear developmental aspect. Clinical features, including cognitive deficits and psychotic phenomena develop over the life span. Symptoms are thought to arise in genetically vulnerable individuals in interaction with environmental, epistatic, and stochastic processes. This aspect of 22q11DS may be highly relevant for the idiopathic schizophrenia syndrome. Approximately 30% of children with a microdeletion of 22q11 will develop a form of schizophrenia that clinically and neurocognitively cannot be distinguished from the idiopathic disorder. Important insights into the trajectory from risk to disorder in idiopathic schizophrenia may be gained from ongoing longitudinal studies of these children comparing cognitive, affective and neural development in those who do and do not develop psychosis among this cohort with a similar genomic deletion [46]. Thus, future research in this field should longitudinally investigate PBMC gene expression in relationship with the phenotypical expression of symptoms in a larger sample of young 22q11DS patients.

## Supporting Information

Table S1 **Full list of transcripts (N = 262) differentially expressed between patients and controls (p<0.05 and Fold Change>1.5).** Columns show (from left to right): Affymetrix Probe Set ID; Gene Abbreviation; Chromosomal location; log-transformed ratio of gene expression patients/controls; significance (not corrected for multiple comparisons: see text methods).
(XLS)
