# Correction: Functional Gene-Expression Analysis Shows Involvement of Schizophrenia-Relevant Pathways in Patients with 22q11 Deletion Syndrome

**DOI:** 10.1371/annotation/d80f4e7d-5e96-41da-9dae-717b0d0d3c60

**Published:** 2012-04-23

**Authors:** Nico J. M. van Beveren, Lianne C. Krab, Sigrid Swagemakers, Gabriëe H.S. Buitendijk, Erik Boot, Peter van der Spek, Ype Elgersma, Therese A. M. J. van Amelsvoort

There was an error in the fourth author's name. The correct name is Gabriëlle H.S. Buitendijk. The correct citation is van Beveren NJM, Krab LC, Swagemakers S, Buitendijk GHS, Boot E, et al. (2012) Functional Gene-Expression Analysis Shows Involvement of Schizophrenia-Relevant Pathways in Patients with 22q11 Deletion Syndrome. PLoS ONE 7(3): e33473. doi:10.1371/journal.pone.0033473 

